# Development of Biomimetic Substrates for Limbal Epithelial Stem Cells Using Collagen-Based Films, Hyaluronic Acid, Immortalized Cells, and Macromolecular Crowding

**DOI:** 10.3390/life14121552

**Published:** 2024-11-26

**Authors:** Mehmet Gurdal, Gulinnaz Ercan, Ozlem Barut Selver, Daniel Aberdam, Dimitrios I. Zeugolis

**Affiliations:** 1 Department of Medical Biochemistry, Faculty of Medicine, Ege University, 35100 Izmir, Türkiye; gulinnaz.ercan@ege.edu.tr; 2 Regenerative, Modular & Developmental Engineering Laboratory (REMODEL) and Science Foundation Ireland (SFI) Centre for Research in Medical Devices (CÚRAM), Biomedical Sciences Building, University of Galway, H91 TK33 Galway, Ireland; dimitrios.zevgolis@ucd.ie; 3 Department of Stem Cell, Institute of Health Sciences, Ege University, 35100 Izmir, Türkiye; ozlem.barut.selver@ege.edu.tr; 4 Department of Ophthalmology, Faculty of Medicine, Ege University, 35100 Izmir, Türkiye; 5 INSERM U1138, Centre des Cordeliers, Université de Paris, 75006 Paris, France; daniel.aberdam@inserm.fr; 6 Regenerative, Modular & Developmental Engineering Laboratory (REMODEL), Charles Institute of Dermatology, Conway Institute of Biomolecular & Biomedical Research and School of Mechanical & Materials Engineering, University College Dublin (UCD), D04 V1W8 Dublin, Ireland

**Keywords:** biomimetic substrates, extracellular matrix deposition, immortalized cells, macromolecular crowding, limbal epithelial stem cells

## Abstract

Despite the promising potential of cell-based therapies developed using tissue engineering techniques to treat a wide range of diseases, including limbal stem cell deficiency (LSCD), which leads to corneal blindness, their commercialization remains constrained. This is primarily attributable to the limited cell sources, the use of non-standardizable, unscalable, and unsustainable techniques, and the extended manufacturing processes required to produce transplantable tissue-like surrogates. Herein, we present the first demonstration of the potential of a novel approach combining collagen films (CF), hyaluronic acid (HA), human telomerase-immortalized limbal epithelial stem cells (T-LESCs), and macromolecular crowding (MMC) to develop innovative biomimetic substrates for limbal epithelial stem cells (LESCs). The initial step involved the fabrication and characterization of CF and CF enriched with HA (CF-HA). Subsequently, T-LESCs were seeded on CF, CF-HA, and tissue culture plastic (TCP). Thereafter, the effect of these matrices on basic cellular function and tissue-specific extracellular matrix (ECM) deposition with or without MMC was evaluated. The viability and metabolic activity of cells cultured on CF, CF-HA, and TCP were found to be similar, while CF-HA induced the highest (*p* < 0.05) cell proliferation. It is notable that CF and HA induced cell growth, whereas MMC increased (*p* < 0.05) the deposition of collagen IV, fibronectin, and laminin in the T-LESC culture. The data highlight the potential of, in particular, immortalized cells and MMC for the development of biomimetic cell culture substrates, which could be utilized in ocular surface reconstruction following further in vitro, in vivo, and clinical validation of the approach.

## 1. Introduction

Cellular therapies have the potential to treat a multitude of currently intractable diseases and have attracted increasing interest in recent years [1,2,3,4,5,6,7]. The transfer of academic knowledge to industry, coupled with a substantial increase in investment within the sector, has enabled the emergence of cell-based therapy products in the marketplace [8,9,10,11,12,13,14,15]. In fact, despite the global market size of cell therapy being estimated at USD 14.52 billion in 2023 and forecast to rise to USD 97 billion by 2033 [16], only a few cell-based tissue-engineered therapy products (Holoclar, cultured autologous corneal epithelial cells containing LESCs attached to a supporting fibrin layer; Spherox, cultured autologous chondrocytes and self-synthesized ECM) have been authorized by the European Medicines Agency (EMA) to date [14]. This is particularly due to the sophisticated processes involved in the manufacture of these products [17,18,19], such as the achievement of in vitro expansion of therapeutic cells (e.g., stem cells) without an unintended phenotypic shift on a biomimetic culture substrate [20,21]. Consequently, over recent decades, research has concentrated on the development of in vitro culture environments [22,23] that facilitate cell expansion, including the use of composite biomaterials [24,25] and self-assembled supramolecular substrates [26,27,28,29].

Tissue engineering by self-assembly [30] is fundamentally based on the use of cells’ intrinsic capacity to create supramolecular substrates (e.g., ECM-enriched matrices) that closely mimic native tissues [31,32,33]. However, the use of conventional culture media requires an extended in vitro cultivation phase for the creation of the transplantable ECM-enriched matrices [34]. Unfortunately, this conventional approach is accompanied by a number of inherent limitations due to the prolonged culture process, including the occurrence of unintended phenotypic drifts and high manufacturing costs [35,36,37,38]. In contrast, macromolecular crowding (MMC) represents a biophysical phenomenon that enhances the rate of biochemical reactions and biological processes [39]. This phenomenon mimics the in vivo density of tissue, resulting in increased and accelerated tissue-specific ECM deposition [40,41,42]. Although MMC, the supplementation of culture media with macromolecules, has been demonstrated to increase and accelerate ECM deposition in a multitude of human primary cell culture systems [43,44,45,46,47], the intrinsic limitations of primary cells [48], including restricted expansion potential [49,50,51], heterogeneity, and inter- and intra-donor variability [52,53,54], render them suboptimal cell sources for the standardizable, scalable, and sustainable manufacture of transplantable ECM-enriched biomaterials. In contrast, immortalized cells offer a promising alternative cell source with the potential to address the aforementioned issues associated with the use of primary cells. This is attributable to their increased expansion capacity and homogeneity [55,56,57,58], which enable them to serve as a standardizable, scalable, sustainable, and consistent cell source for the manufacture of tissue-engineered products.

Collagen, an indispensable structural component of the ECM [59,60], is a popular biomaterial due to its natural origin. It is, therefore, employed extensively in the development of tissue-engineered biomaterials, including those used in corneal bioengineering [61,62,63]. In recent years, there has been another increasing interest in polysaccharide-enriched biomaterials [64,65] due to the fact that polysaccharides are an essential component of the native microenvironments, exerting a regulatory effect on cellular functions through the facilitation of cell–cell and cell–matrix interactions [66,67]. For instance, HA constitutes a significant proportion of the glycosaminoglycan (GAG) content of biological fluids [68,69,70,71,72,73] and various solid tissues [74,75,76,77,78], including the cornea, particularly the basal epithelium of the limbus, which is the niche environment for LESCs [79,80,81]. Indeed, the presence of HA in the LESCs niche is necessary for the maintenance of LESCs in their “stem cell” state [82], thereby preventing them from undergoing unintended differentiation.

LSCD is defined by the loss of LESCs, which are situated in the limbal basal epithelium and are essential for the corneal epithelial regeneration following injury to the cornea (e.g., chemical or thermal burns) [83,84,85]. Although among the therapeutic strategies, the transplantation of in vitro expanded LESCs on a carrier substrate, such as human amniotic membrane (hAM), is the most promising approach [86,87,88], the use of hAM is hindered by several factors, including a lack of standardization due to inter- and intra-donor variability, limited availability, and potential infection risks [89,90,91,92,93]. In fact, hAM serves as a natural model for the native microenvironment due to the inherent structural components it contains, including collagens (e.g., collagen type I and IV), non-collagenous glycoproteins (e.g., laminin and fibronectin), and GAGs (e.g., HA) [94,95,96,97], which collectively contribute to the maintenance of the native limbal niche [98,99]. However, the development of an alternative culture substrate that can replace hAM remains an ongoing challenge. While the combination of collagen-based materials with GAGs (e.g., HA) represents a promising approach, the incorporation of additional ECM components (e.g., collagen IV, fibronectin, and laminin) into the model utilizing the intrinsic capacity of the cells would increase biomimicry and thereby more closely resemble the LESCs niche. Nevertheless, the formation of such ECM-rich supramolecular surrogates through the use of traditional dilute culture milieu requires an extended in vitro cultivation phase (e.g., 35 days for cornea, 50 days for skin, 130 days for cartilage, and 168 days for blood vessel) [34].

With these in mind, herein we ventured to develop biomimetic culture substrates for LESCs using in-house-extracted collagen to fabricate collagen-based films, HA to enrich collagen-based films, immortalized LESCs as the cell source to produce native-like ECM components, and MMC to increase and accelerate ECM deposition on the collagen-based films. Overall, this study introduced an innovative approach combining the effectiveness of collagen-based films, HA, and MMC with immortalized cells to develop novel tissue-engineered products for ocular surface reconstruction.

## 2. Materials and Methods

### 2.1. Materials

Bovine Achilles tendons employed in this study were sourced from steers with an age of 24 months, which were slaughtered at a local abattoir. Four-arm PEG succinimidyl glutarate (4SP; Mw 10 kDa), HA (Mw 1000 kDa), Ficoll™ (Mw 1000 kDa) and λ-carrageenan (Viscarin^®^ GP 209 NF) were procured from JenKem Technology (Plano, TX, USA), Lifecore Biomedical (Chicago, IL, USA), TdB Consultancy (Uppsala, Sweden) and FMC International Health and Nutrition (Dublin, Ireland), respectively. Tissue culture materials were procured from Sarstedt (Dublin, Ireland). Unless otherwise indicated, all other materials and reagents were procured from Sigma-Aldrich (Dublin, Ireland).

### 2.2. Collagen Extraction and Collagen Film Fabrication

#### 2.2.1. Type I Collagen Extraction

The collagen extraction process was conducted in accordance with established protocols [100,101], with minor modifications. The connective tissue encasing the tendons was manually separated and pulverized with a cryomill (Freezer/Mill 6870, SPEX SamplePrep, Metuchen, NJ, USA) prior to undergoing a washing process utilizing 1X phosphate-buffered saline (PBS). The ground tendon tissue was subjected to dissolution in 1 M acetic acid on an orbital shaker for 48 h at 4 °C. Thereafter, the solution was supplemented with pepsin at a dosage of 80 units per milligram. The solution was mixed and incubated for a period of 72 h at 4 °C. The insoluble tendon was dissociated by filtration and centrifugation (20 min at 21,000× *g*, 4 °C), thereby facilitating the collection of the soluble collagen. Subsequently, collagen purification was conducted via salt precipitation at a specific salt concentration of 0.9 M NaCl, which precipitates collagen type I. The precipitated collagen was collected following centrifugation (20 min at 21,000× *g*, 4 °C) and then re-suspended in 1 M acetic acid. The collagen solution was subjected to repeated dialysis with a molecular weight cut-off of 8000 against 1 mM acetic acid. The resulting collagen solution was lyophilized through a freeze-dryer (VirTis Advantage 2.0 Benchtop Freezer Dryer, Scientific Products, Warminster, PA, USA). Subsequently, the lyophilized collagen sponges were preserved at −80 °C for subsequent analyses. The purity and quality of the in-house-extracted collagen were analyzed via gel electrophoresis and collagen solubility assessment, as has been previously detailed [102]. The solubility of the collagen extracts was evaluated through visual inspection of the solution after centrifugation at 13,800× *g* for 15 min at 4 °C, which is regarded as a qualitative technique. Moreover, a consistent concentration (5 mg/mL) of collagen was employed throughout the production of the collagen films. Prior to the fabrication process, the collagen concentration was quantified through a hydroxyproline assay, as has been previously detailed [102].

#### 2.2.2. Collagen Film Fabrication

The fabrication of the collagen film was conducted in accordance with the typical protocol [103], with minor modifications. The freeze-dried collagen and HA were separately reconstituted in 0.05 M acetic acid and 1X PBS, respectively, using a medium magnetic stirrer over the course of one night. The pH value of the homogeneous collagen solutions was calibrated to a range of between 5.0 and 6.0 by the addition of a concentrated solution of sodium hydroxide (NaOH). The collagen solution was enriched with 0.5% HA (*w*/*w*). An additional collagen solution, which did not undergo HA enrichment, was prepared for use as a control. The final collagen concentration in the solutions was maintained at 5 mg/mL. The resulting solutions were then mixed until a homogeneous mixture was achieved. The air bubbles were removed by subjecting the solution to a centrifugal force (2 min at 360× *g*). The final mixtures were pipetted at a constant volume of 250 μL/cm^2^ into the wells of cell culture plates (e.g., 24- or 48-well plates) or onto a surface suitable for the analysis to be performed in order to achieve a uniform thickness across all samples. The samples were then transferred to a laminar flow cabinet and subjected to evaporation until the formation of films was complete. Subsequently, the surfaces of the resulting films were coated with increasing concentrations (1, 2.5, 7.5, and 15 mM) of 4SP solution (in 1X PBS). The 4SP solution was incubated overnight at room temperature in a laminar flow cabinet on the films to ensure cross-linking of collagen molecules. In addition, 0.625% GTA in 1X PBS standard solution was employed for GTA cross-linking [104]. The films were then allowed to dry in the chamber after a brief washing with 1X PBS. Sterilization of the produced films was achieved through UV.

### 2.3. Characterization of Fabricated Collagen Films

#### 2.3.1. Quantification of Free Amine Groups

In this study, the free amine groups of collagen molecules were evaluated by tri-nitrobenzene sulphonic acid (TNBSA) assay in accordance with the established protocol, with minor modifications [102]. A total of 1 mg of collagen film samples to be analyzed was placed in 2 mL microtubes, with at least three replicates per sample. Subsequently, 500 µL of a 0.1 M sodium bicarbonate solution was added to the tubes. To construct the standard curve, glycine solutions were prepared as follows: 0, 0.005, 0.01, 0.02, 0.03, 0.04, and 0.05 mg/mL glycine (Fisher BioReagents, Dublin, Ireland) in 0.1 M sodium bicarbonate. A total of 500 μL of each prepared standard curve solution was transferred into 2 mL tubes, with at least three replicates per standard. A volume of 250 μL of the 0.01% (*w*/*v*) TNBSA (Thermo Fisher Scientific, Dublin, Ireland) in 0.1 M sodium bicarbonate was then added to the tubes containing the samples and standards. The tubes were mixed by vortexing and then incubated at 37 °C with approximately 150 rpm agitation for one hour, with the tubes protected from light using aluminum foil. Subsequently, 250 μL of 10% (*w*/*v*) sodium dodecyl sulfate (SDS) in deionized water and 125 μL of 1 M HCl were added to the tubes, respectively. Thereafter, the tubes containing the collagen film samples were incubated at 120 °C for 15–20 min (until no insoluble film remained in the tubes). A total of 100 μL of the solutions in the tubes were transferred to 96-well microplates. Following the careful removal of any bubbles formed due to SDS using needles, a spectrophotometric measurement was conducted at a wavelength of 335 nm using a microplate reader (Varioskan Flash, Thermo Fisher Scientific, Dublin, Ireland).

#### 2.3.2. Degradation Assay (Collagenase Assay)

The stabilization level of cross-linked collagen was also evaluated by determining the resistance of collagen molecules to enzymatic degradation by bacterial collagenase. The weight of each empty microtube was recorded with a precision scale. A minimum of 5 mg of the fabricated collagen film samples (with a minimum of three replicates) was placed into each tube. Subsequently, the tubes were weighed once more, and the results were recorded. A solution of 1 mL of collagenase I (at a concentration of 50 U/mL) in lysis buffer (prepared in 0.1 M Tris-HCl) was then added to the tubes. Following this, the tubes were incubated at 37 °C with approximately 150 rpm agitation for analysis at specified time points (0, 12, 24, and 48 h). Thereafter, the samples were subjected to centrifugation at 3500× *g* for 10 min, following the incubation periods. The supernatant was aspirated with great care, ensuring that no contact was made with the sediment at the bottom of the tube. A total of 1 mL of deionized water was then added to the tubes, and the samples were centrifuged a second time at 3500× *g* for 10 min. Following the removal of the supernatant, the tubes were stored at −80 °C overnight. The remaining liquid in the tubes was then completely dried using the freeze-dryer, which took at least 24 h. After the lyophilization, the tubes were weighed once more, and the data were recorded. The weights of the collagen films that remained intact in the tubes were calculated [102].

#### 2.3.3. Quantification of Denaturation Temperature

Quantification of the denaturation temperature of the fabricated collagen films was conducted via a differential scanning calorimeter (DSC-60, Shimadzu, Kyoto, Japan), as previously detailed [105]. The fabricated collagen films were initially subjected to an overnight incubation period in 1X PBS. Thereafter, the samples were promptly blotted utilizing filter paper with the objective of removing surface or unbound water. The subsequent step involved sealing the samples in aluminum pans and applying a uniform heating ramp at a rate of 5 °C/min over a temperature range of 25 to 90 °C. An empty pan was employed as a control. The denaturation temperature was identified to be the peak of the endothermic curve, representing the maximum heat absorption observed.

### 2.4. Cell Culture

T-LESCs, obtained from D. Aberdam’s laboratory (Paris, France), were maintained and expanded using keratinocyte serum-free medium (K-SFM) (Gibco, Life Technologies, Dublin, Ireland) supplemented with 25 μg/mL bovine pituitary extract (Gibco, Life Technologies, Dublin, Ireland), 0.2 ng/mL epidermal growth factor, 0.4 mM calcium chloride, 2 mM l-glutamine, and 100 U/mL penicillin/streptomycin at 37 °C in a humidified atmosphere of 5% CO_2_, as previously described [106,107].

### 2.5. Macromolecular Crowding (MMC)

The cells were seeded directly into 24-well tissue culture plates or into 24-well plates containing fabricated collagen films, as detailed in Section 2.2.2. The seeding density was 25,000 cells/cm^2^. On a subsequent day, the medium was refreshed with the maintenance medium supplemented with 100 µM l-ascorbic acid 2-phosphate sesquimagnesium salt hydrate in the absence (−MMC) or in the presence of MMC. In the present study, 10 µg/mL λ-carrageenan (λ CR; previously optimized in human umbilical cord mesenchymal stromal cell cultures [108]) and a Ficoll™ cocktail (FC; 10 mg/mL Ficoll™ 70 kDa, 25 mg/mL Ficoll™ 400 kDa, 2.25 mg/mL Ficoll™ 1000 kDa; previously optimized in human dermal fibroblast [109] and human bone marrow mesenchymal stromal cell [110] cultures) were used as MMC agents. The data were collected at three designated time points: day 4, day 7, and day 10. 

### 2.6. Basic Cell Function Analysis

At each designated time point, days 4, 7, and 10, cellular morphology was evaluated via phase-contrast microscopy. Image acquisition was conducted using an inverted microscope (Leica Microsystems, Wetzlar, Germany), and the subsequent data processing was performed using the LAS EZ 2.0.0 software. Phase-contrast microscopy was employed exclusively for the cells cultured on TCP, as the collagen films did not yield sufficiently clear images for the morphological analysis of the cultured cells. Therefore, images for cellular viability (obtained via fluorescence microscopy) were used to evaluate the cellular morphology of the cells cultured on the collagen films.

The viability of the cells was assessed using the Live/Dead^®^ viability kit (Invitrogen™, Thermo Fisher Scientific, Dublin, Ireland) in accordance with the instructions provided by the manufacturer at each designated time point (days 4, 7, and 10). In brief, the cells were incubated in a solution of calcein AM (2 mM) and ethidium homodimer (4 mM) in Hanks’ balanced salt solution (HBSS). Imaging was conducted using an Olympus IX-81 inverted fluorescence microscope (Olympus Corporation, Tokyo, Japan) with a FITC filter for calcein AM and a Texas Red filter for ethidium homodimer-1. The green-colored cells were deemed viable, whereas the red cells were considered non-viable.

In order to determine cellular proliferation, DNA quantification was conducted at each designated time point (days 4, 7, and 10) utilizing the Quant-iT™ PicoGreen^®^ dsDNA assay kit (Invitrogen™, ThermoFisher Scientific, Dublin, Ireland) in accordance with the instructions provided by the manufacturer. The DNA was extracted through the implementation of three freeze–thaw cycles, with the incorporation of 200 µL of nucleic acid-free water per cell culture well. The standard curve solutions were prepared in a 96-well plate containing 1x TE buffer. Subsequently, 100 μL of each sample was transferred to the 96-well plate, followed by the addition of 100 μL of diluted PicoGreen^®^ to each well. Following a 5 min incubation period at room temperature in the absence of light, the plate was evaluated through the use of the microplate reader with an excitation wavelength of 480 nm and an emission wavelength of 525 nm.

To assess cellular metabolic activity, the alamarBlue^®^ kit (Invitrogen™, ThermoFisher Scientific, Dublin, Ireland) was employed at each designated time point (days 4, 7, and 10) in compliance with the manufacturer’s instructions. The samples were incubated in HBSS comprising 10% alamarBlue^®^ reagent, following the removal of the culture medium and a rinsing step with HBSS. This was conducted to permit the resazurin dye reduction by active cells in the cell metabolism assay, which was then incubated for 4 h at 37 °C. Subsequently, 100 µL of the samples were pipetted into a 96-well plate, and the absorbance was read at 550 nm and 595 nm through the use of the microplate reader. The percentage of alamarBlue^®^ reduced (AR) by the cells was calculated in accordance with the manufacturer’s protocol using the following formulation: AR = [A_LW_ − (A_HW_ × CF)] × 100. In this formula, A_LW_ represents the absorbance at the lower wavelength (550 nm), A_HW_ represents the absorbance at the higher wavelength (595 nm), and CF represents the correlation factor. The CF is defined as the ratio of the differences between the absorbances of alamarBlue^®^ and HBSS alone at the two wavelengths (550 and 595 nm). Consequently, the cellular metabolic activity was expressed as a % reduction in the alamarBlue^®^ and was normalized to the control groups.

### 2.7. SDS-PAGE for Collagen Type I Deposition

The gel electrophoresis was performed in accordance with the previously established protocol [44]. At each designated time point (days 4, 7, and 10), the media were removed, and following rinsing via HBSS, the cell layers were subjected to a 2 h digestion process utilizing pepsin derived from porcine gastric mucosa at 37 °C with continuous agitation using a shaker. Subsequently, the cell layers were subjected to a scratch using 1 mL pipette tips, followed by neutralization with 1 N NaOH. The samples to be analyzed by sodium dodecyl sulfate–polyacrylamide gel electrophoresis (SDS-PAGE) were appropriately diluted (15:1) with deionized water and 5X sample buffer. A 10 μL sample solution was introduced into the designated wells of the gel (comprising 5% separation gel and 3% stacking gel), following a 5 min incubation period at 95 °C. The electrophoresis was conducted using a Mini-PROTEAN Tetra Electrophoresis System (Bio-Rad, Dublin, Ireland). The initial potential difference applied was 50 mV, which was maintained for the first 30 min. Thereafter, the potential difference was increased to 120 mV, which was maintained for the remainder of the process. The gels were then washed in a gentle manner with ultra-pure water and stained through the utilization of the SilverQuest™ Silver Staining Kit (Invitrogen™, ThermoFisher Scientific, Dublin, Ireland) in accordance with the manufacturer’s instructions. Subsequently, the gels were imaged using a gel scanner following a quick rinse using water. To quantitatively assess the deposition of collagen type I on the cell layers, the relative density of the collagen a1(I) and a2(I) chains was measured and then analyzed in comparison to the density of the a1(I) and a2(I) chain bands of the commercial collagen type I standard purchased from Symatese Biomateriaux, Chaponost, France. The SDS-PAGE for the deposition of collagen type I was conducted solely on the cells cultured on TCP, as the deposited collagen was not detectable on the gels. SDS-PAGE was, therefore, not utilized for subsequent analyses.

### 2.8. Immunofluorescence Analysis

At each designated time point (days 4, 7, and 10), cell layers were subjected to three 5 min washes in 500 µL of HBSS. Thereafter, the samples were fixed using a pre-cold (4 °C) 4% paraformaldehyde (PFA) fixative for a period of 15 min at room temperature. In the event that the protein of interest was located intracellularly, the samples underwent a permeabilization process involving Triton X-100 (0.2% *v*/*v*), which facilitated the permeation of the cell membrane. Following three washes in 1X PBS, the samples were incubated in 3% bovine serum albumin (BSA) for a period of 30 min at room temperature with the purpose of blocking non-specific binding. Subsequently, the cells underwent overnight incubation at 4 °C in the presence of one of the following primary antibodies or in the absence of any antibody, in which case PBS served as the negative control. Rabbit anti-collagen type I (1:200 dilution in PBS, Boster, Oxfordshire, UK); rabbit anti-collagen type IV (1:200 dilution in PBS, Abcam, Cambridge, UK); rabbit anti-fibronectin (1:200 dilution in PBS, Abcam, Cambridge, UK); rabbit anti-laminin (1:200 dilution in PBS, Abcam, Cambridge, UK); rabbit anti-paired box 6 (PAX6; 1:200 dilution in PBS, Abcam, Cambridge, UK); rabbit anti-vimentin (1:1000 dilution in PBS, Abcam, Cambridge, UK); rabbit anti-cytokeratin 12 (CK12; 1:200 dilution in PBS, Abcam, Cambridge, UK). Subsequent to three washes of the samples with PBS, the bound primary antibodies were visualized using either AlexaFluor^®^ 488 donkey anti-mouse (1:400 dilution in PBS, ThermoFisher Scientific, Dublin, Ireland) or AlexaFluor^®^ 488 goat anti-rabbit (1:400 dilution in PBS, ThermoFisher Scientific, Dublin, Ireland) secondary antibodies for one h at room temperature. The unbound antibodies were then rinsed on three occasions with PBS, after which Hoechst 33342 (1:2000 dilution in PBS, Invitrogen™, ThermoFisher Scientific, Dublin, Ireland) was introduced in order to stain the cell nuclei. The images were captured via the inverted fluorescence microscope. The fluorescence intensities were then quantified in a minimum of three areas per experimental sample through the utilization of ImageJ software (https://imagej.net/ij/, NIH, Bethesda, MD, USA). The fluorescence intensities were calculated as a percentage difference between the treated cells and the control cells (−MMC). To ensure accurate comparisons between the groups, the calculated relative fluorescence intensities were normalized to the respective cell numbers.

### 2.9. Statistical Analysis

The data were represented as mean ± standard deviation. Each experiment was carried out in triplicate at a minimum. The statistical analysis was conducted with the use of the IBM^®^ SPSS^®^ Statistics 26 software. A one-way analysis of variance (ANOVA) was employed for multi-comparative analyses, with a least significant difference (LSD) *post hoc* test conducted for pairwise comparative analyses. This was conducted following confirmation of the samples’ normal distribution (Kolmogorov–Smirnov test) and equal variances (Levene’s test for homogeneity of variances). In instances where either or both of the aforementioned conditions were not met, a non-parametric analysis was performed via the Kruskal–Wallis test for the comparison of multiple groups and the Mann–Whitney test for the comparison of two groups. Statistical significance was determined at a *p*-value of less than 0.05 for differences between selective experimental groups.

## 3. Results

### 3.1. Characterization of Fabricated Collagen Films

The in-house-extracted collagen type I displayed the standard electrophoretic mobility profile (Figure S1A) and similar solubility (Figure S1B), comparable to that of commercially available preparations.

**Figure 1:**
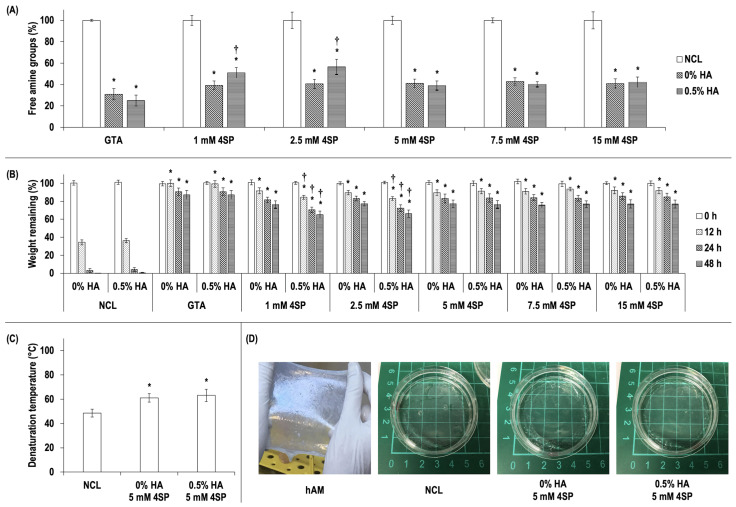
Characterization of fabricated collagen films. (**A**) Quantification of free amine groups (TNBSA assay) of collagen films cross-linked with GTA (as a positive control) and with increasing concentration of 4SP. (**B**) Degradation analysis by collagenase assay. (**C**) Denaturation temperature measured by differential scanning calorimetry (DSC); (**D**) macroscopic observation of the transparency of the fabricated films in comparison to hAM. The image of hAM was sourced directly from the archive of Dr. Ozlem Barut Selver. * indicates a statistically significant (*p* < 0.05) difference compared to NCL. † indicates a statistically significant (*p* < 0.05) difference between collagen films in the absence of HA (0% HA) and in the presence of HA (0.5% HA) at a given 4SP concentration group. N = 3.

GTA, recognized as a potent collagen cross-linker, was utilized as a positive control in this study to assess the cross-linking efficiency of collagen molecules. The TNBSA assay (Figure 1A) revealed that the cross-linking of the collagen films using GTA in the absence of HA or in the presence of 0.5% HA resulted in significantly (*p* < 0.05) lower free amine groups in comparison to non-cross-linked collagen films (NCL); no significant (*p* > 0.05) differences in free amine groups were detected between the GTA-cross-linked collagen films in the absence of HA and in the presence of 0.5% HA. The collagen films that had been cross-linked with each of the five concentrations of 4SP (1, 2.5, 5, 7.5, and 15 mM) yielded significantly (*p* < 0.05) lower free amine groups in comparison to the NCL. The 4SP concentration of 5 mM and above did not result in any statistically significant (*p* > 0.05) differences in free amine groups between the cross-linked collagen films in the absence of HA and in the presence of 0.5% HA; however, collagen films enriched with 0.5% HA and cross-linked with the concentration of 1 mM and 2.5 mM 4SP resulted in significantly (*p* < 0.05) higher free amine groups compared to collagen films cross-linked with the same concentrations (1 mM and 2.5 mM) of 4SP in the absence of HA.

The in vitro enzymatic degradation assay (Figure 1B) demonstrated that NCL films were degraded almost entirely within 24 h. In contrast, GTA and 4SP (all concentrations tested) exhibited significantly (*p* < 0.05) higher resistance to enzymatic degradation than NCL groups. The 4SP concentration of 5 mM and above did not result in any statistically significant (*p* > 0.05) differences in resistance to enzymatic degradation between the cross-linked collagen films in the absence of HA and in the presence of 0.5% HA; however, collagen films enriched with 0.5% HA and cross-linked with the concentration of 1 mM and 2.5 mM 4SP resulted in significantly (*p* < 0.05) lower resistance to enzymatic degradation compared to collagen films cross-linked with the same concentrations (1 mM and 2.5 mM) of 4SP in the absence of HA.

The TNBSA (Figure 1A) and collagenase (Figure 1B) assays revealed that HA enrichment affected the cross-linking efficiency of 4SP at concentrations of 1 mM and 2.5 mM in the collagen films, whereas HA enrichment did not affect the cross-linking efficiency of 4SP at concentrations of 5 mM and above in the collagen films. It was thus determined that a 5 mM concentration of 4SP represents the lowest effective concentration for the cross-linking of collagen films enriched with 0.5% HA. Consequently, subsequent experiments were conducted with a 5 mM concentration of 4SP.

The differential scanning calorimetry (DSC) assessment (Figure 1C) demonstrated that 4SP at a concentration of 5 mM significantly (*p* < 0.05) increased the denaturation temperature of the collagen films in comparison to NCL, whereas no significant (*p* > 0.05) differences in the denaturation temperature were detected between the 4SP-cross-linked collagen films in the absence of HA and in the presence of 0.5% HA.

A macroscopic analysis of the transparency (Figure 1D) was conducted through visual inspection and thus considered to be a qualitative technique. The macroscopic analysis of the transparency demonstrated that non-cross-linked collagen films (NCL), 4SP-cross-linked collagen films (CF), and 4SP-cross-linked collagen films enriched with HA (CF-HA) were transparent and colorless in appearance. In contrast, hAM (image source is Dr. Ozlem Barut Selver’s archive) exhibits an opaque appearance.

### 3.2. Efficacy of MMC Agents in T-LESCs Culture on TCP

**Figure 2:**
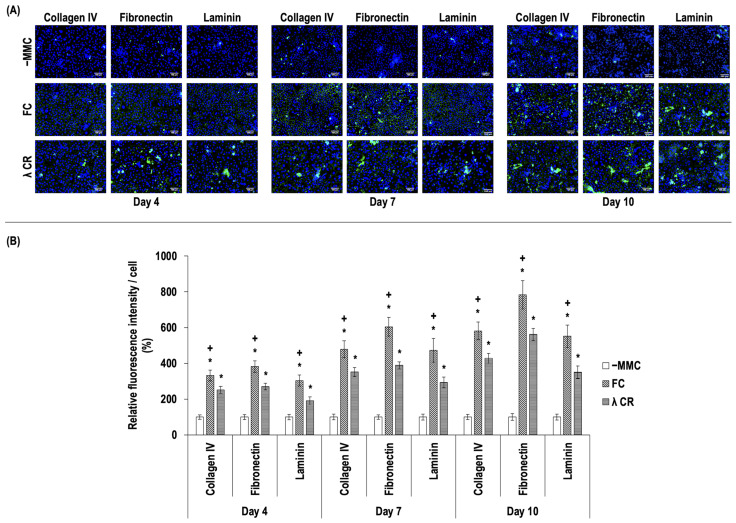
Immunofluorescence (**A**) and complementary relative fluorescence intensity analysis (**B**) of deposited collagen type IV, fibronectin, and laminin at days 4, 7, and 10 in T-LESC cultures on TCP without MMC (−MMC) and with MMC (FC and λ CR). T-LESCs: human telomerase-immortalized limbal epithelial stem cells; TCP: tissue culture plastic; MMC: macromolecular crowding; FC: Ficoll™ cocktail; CR: carrageenan. * indicates a statistically significant (*p* < 0.05) difference compared to −MMC. + indicates the highest (*p* < 0.05) population in the respective ECM molecule at a given time point. N = 9. Collagen type IV, fibronectin, and laminin: Green. Nuclei: Blue. Scale bar: 100 µm.

In this study, the efficacy of FC and λ CR as MMC agents in T-LESC culture on TCP was initially evaluated. T-LESCs exhibited a characteristic cobblestone morphology, independent of the absence or presence of FC and λ CR (Figure S2A). The qualitative cellular viability analysis showed no differences between the groups (−MMC, FC, and λ CR) at each time point (days 4, 7, and 10) (Figure S2B). No significant (*p* > 0.05) differences in cellular metabolic activity (Figure S2C) and proliferation, as assessed by DNA quantification (Figure S2D), were detected between the groups at a given time point. SDS-PAGE (Figure S3A) and immunofluorescence (Figure S3B) analyses revealed no collagen type I deposition in the T-LESC cultures on TCP without MMC and with MMC (FC and λ CR). Immunofluorescence and complementary relative fluorescence intensity analysis (Figure 2) revealed that the FC and the λ CR induced significantly (*p* < 0.05) higher collagen type IV, fibronectin, and laminin deposition compared to the −MMC group at all time points (days 4, 7 and 10). At each time point, immunofluorescence (Figure 2A) and complementary relative fluorescence intensity analysis (Figure 2B) revealed that FC induced significantly (*p* < 0.05) the highest collagen type IV, fibronectin, and laminin deposition among the groups (−MMC, FC, and λ CR). As the FC was identified as the most efficacious MMC agent for T-LESC cultures on TCP among the agents tested in this study, subsequent assessments were conducted using only the FC.

### 3.3. Efficacy of MMC in T-LESC Cultures on Fabricated Collagen Films

**Figure 3:**
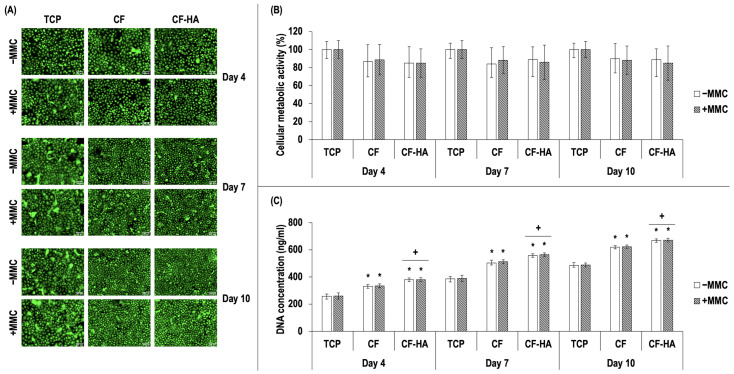
Viability (**A**), metabolic activity (**B**), and DNA concentration (**C**) at days 4, 7, and 10 in T-LESC cultures on TCP, CF, and CF-HA without MMC (−MMC) and in the presence of the FC (+MMC). The cellular metabolic activity was expressed as a % reduction in the alamarBlue^®^ and was normalized to the TCP control group. T-LESCs: human telomerase-immortalized limbal epithelial stem cells; TCP: tissue culture plastic; CF: collagen films; CF-HA: collagen films enriched with HA; MMC: macromolecular crowding. * indicates a statistically significant (*p* < 0.05) difference in comparison to TCP at a given time point. + indicates the highest (*p* < 0.05) population among the tested culture substrates at a given time point. N = 3. Live cells: Green. Dead cells: Red. Scale bar: 100 µm.

Starting with basic cell function (morphology, viability, metabolic activity, and proliferation) analysis, at each time point (days 4, 7, and 10), T-LESCs maintained their characteristic cobblestone morphology on the collagen films, independent of the absence or presence of MMC (Figure 3A). Qualitative cellular viability analysis showed no differences between the groups at each time point (Figure 3A). No statistically significant (*p* > 0.05) differences in cellular metabolic activity were detected between the groups at a given time point (Figure 3B). With regard to the cell proliferation, as determined by DNA quantification, CF and CF-HA exhibited significantly (*p* < 0.05) higher DNA content compared to the TCP group at each time point, independent of the absence or presence of MMC and CF-HA exhibited significantly (*p* < 0.05) the highest DNA among the tested culture substrates (TCP, CF, and CF-HA) at each time point, independent of the absence or presence of MMC (Figure 3C).

**Figure 4:**
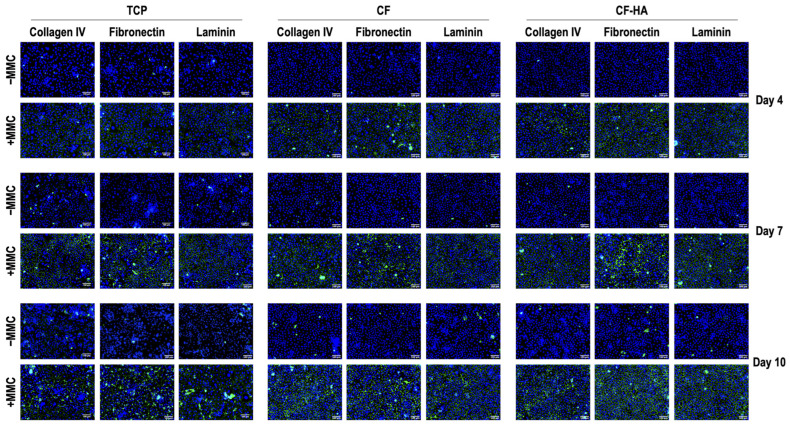
Immunofluorescence analysis of deposited collagen type IV, fibronectin, and laminin at days 4, 7, and 10 in T-LESC cultures on TCP, CF, and CF-HA without MMC (−MMC) and in the presence of the FC (+MMC). T-LESCs: human telomerase-immortalized limbal epithelial stem cells; TCP: tissue culture plastic; CF: collagen films; CF-HA: collagen films enriched with HA; MMC: macromolecular crowding. Collagen type IV, fibronectin, and laminin: Green. Nuclei: Blue. Scale bar: 100 µm.

**Figure 5:**
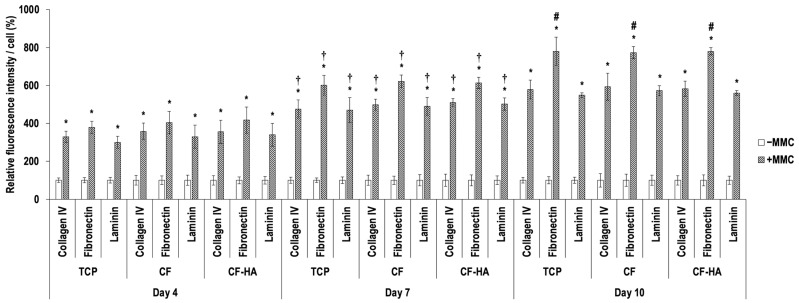
Complementary relative fluorescence intensity analysis of deposited collagen type IV, fibronectin, and laminin normalized to cell number (%) at days 4, 7, and 10 in T-LESC cultures on TCP, CF, and CF-HA without MMC (−MMC) and in the presence of the FC (+MMC). T-LESCs: human telomerase-immortalized limbal epithelial stem cells; TCP: tissue culture plastic; CF: collagen films; CF-HA: collagen films enriched with HA; MMC: macromolecular crowding. * indicates a statistically significant (*p* < 0.05) difference compared to −MMC. † indicates a statistically significant (*p* < 0.05) difference between day 4 and day 7 in a given group. # indicates a statistically significant (*p* < 0.05) difference between day 7 and day 10 in a given group. N = 9.

Immunofluorescence (Figure 4) and complementary fluorescence intensity analysis (Figure 5) revealed that MMC significantly (*p* < 0.05) enhanced the deposition of collagen type IV, fibronectin, and laminin in comparison to the −MMC group at each time point (days 4, 7, and 10), independent of the culture substrates (TCP, CF, and CF-HA). In the presence of MMC, the deposition of collagen IV, fibronectin, and laminin at day 7 was significantly (*p* < 0.05) higher than at day 4, independent of the culture substrates. In the presence of MMC, the deposition of fibronectin at day 10 was significantly (*p* < 0.05) higher than at day 7, independent of the culture substrates. Further immunofluorescence (Figure S4) and complementary relative fluorescence intensity analysis (Figure S5) made apparent no significant (*p* > 0.05) differences between the groups with regard to the staining of PAX6, vimentin, and CK12 molecules at any time point.

## 4. Discussion

Cellular therapies are administered through the injection of therapeutic cells into the patient or the implantation of a biomaterial that carries the therapeutic cells. The direct cell injection is associated with serious disadvantages, including low cell survival and improper localization within the body [111,112,113,114]. Conversely, the implantation of cells attached to biomaterials within the body has the potential to address the drawbacks associated with direct injection. However, the fabrication processes of implantable tissue-engineered devices present a number of significant challenges that require resolution. It is, therefore, imperative to develop approaches that can address the challenges associated with the manufacturing of implantable tissue-engineered devices, including poor mimicking of the native microenvironment, the limited availability of cell sources, and the prolonged, non-standardizable, unscalable, and unsustainable production processes. In this study, we aimed to develop biomimetic substrates using collagen, hyaluronic acid (HA), immortalized LESCs (T-LESCs), and macromolecular crowding (MMC).

### 4.1. Physicochemical Characterization of Fabricated Collagen Films

Collagen, one of the most prevalent structural proteins in human tissues, plays a pivotal role in the extracellular matrix (ECM), providing a supporting framework for cellular attachment due to its natural structure [115]. Given its biocompatible and non-immunogenic properties, collagen is a preferred component in the development of tissue scaffolds and cellular carriers in cell-based tissue engineering research [116]. Additionally, collagens are combined with other molecules in the design of tissue-specific scaffolds [117]. These composite materials exhibit advantageous properties with regard to their physical characteristics and biological responses [118]. However, given that collagens possess limited mechanical strength and undergo rapid degradation, cross-linking techniques are employed to enhance the stability of collagen-based substrates. The mechanical stability and degradation rates of collagen-based biomaterials are regulated by the application of chemical cross-linking methods. Nevertheless, the use of conventional chemical cross-linking techniques, such as glutaraldehyde (GTA) [119,120], has been demonstrated to lead to a number of adverse effects, including calcification [121,122], cytotoxicity [123], foreign body response [124], predominant pro-inflammatory macrophage response, and elevated pro-inflammatory cytokine expression [125,126,127]. Consequently, the in vivo utilization of such traditional cross-linking agents is limited [128]. In contrast, the use of polyethylene glycol (PEG) polymers as the cross-linker agent for the stabilization of biomaterials appears to be promising in terms of their biocompatibility [129,130,131]. In the present study, HA-enriched collagen films were fabricated by cross-linking the collagen molecules using 4SP as the cross-linking agent, which has previously been demonstrated to possess low toxicity [132,133,134]. The physicochemical characterization of the produced HA-enriched collagen films, including the analysis of free amine groups, enzymatic degradation, and denaturation temperature, demonstrated that 4SP effectively crosslinked collagen molecules. Moreover, the TNBSA and collagenase assays indicated that 5 mM was the minimal effective 4SP concentration for cross-linking collagen molecules in HA-enriched collagen films. Although previous studies [135,136] have demonstrated the efficacy of 1 mM 4SP in cross-linking collagen molecules, the observed outcome (5 mM) in the presence of HA molecules is consistent with expectations. This is because it is postulated that the HA molecules may affect the cross-linking activity of 4SP on collagen molecules. The macroscopic observation of the collagen films fabricated in the current study revealed a structure that was more transparent than that of the hAM. Previous studies have also demonstrated that four-arm PEG, when employed as a cross-linking agent for the fabrication of biomaterials, results in transparent structures, which aligns with our observation [135,137,138,139]. In light of the presented evidence, it can be proposed that 4SP has the potential to serve as a promising cross-linker, particularly in the development of biomaterials designed for use in corneal applications.

### 4.2. Efficacy of MMC Agents in T-LESC Culture on TCP

In this study, the efficacy of macromolecular crowders was initially evaluated using T-LESCs cultured on TCP. Starting with cytocompatibility, no notable difference was observed between −MMC and the tested crowding agents with respect to cell viability, metabolic activity, and proliferation (DNA concentration). Moreover, cell growth was clearly detected in all groups. With regard to the deposition of ECM, the presence of collagen type I could not be detected by SDS-PAGE and immunofluorescence analysis. This observation is not surprising, considering that the basement membrane of the limbal epithelium is predominantly comprised of collagen type IV, fibronectin, and laminin [98,140,141]. It is also important to highlight that while epithelial cells are known to secrete unique ECM components in specific quantities into their surrounding environment [142,143,144], fibroblasts are the primary cells responsible for the production and secretion of ECM components in the cornea [145,146,147,148]. The immunofluorescence analysis showed that each MMC agent tested in the current study increased the deposition of collagen IV, fibronectin, and laminin in the cell layer of T-LESCs. Indeed, these proteins are crucial for LESC niche function, including the regulation of LESCs via signal transduction pathways [149]. Furthermore, previous studies have demonstrated that culture substrates formulated with collagen IV, laminin, and fibronectin facilitate the adhesion and growth of LESCs in vitro [150,151,152,153,154,155,156]. With these in mind, our observation regarding the increase in deposition of ECM in the developed culture substrate is highly promising, as it shows that supramolecular assemblies of collagen IV, fibronectin, and laminin can be formed using T-LESCs and MMC, thereby increasing the biomimicry of the unique native limbal microenvironment.

The phenomenon of MMC is a biophysical technique that involves the incorporation of macromolecules into reaction or culture media. It is based on the principles of the excluded volume effect, which states that two molecules cannot simultaneously occupy the same space. MMC significantly decreases molecular diffusion and dramatically enhances the rates and kinetics of biochemical reactions and biological processes (e.g., protein interactions and enzymatic reactions), resulting in increased ECM deposition [34,39]. Despite evidence that MMC enhances and accelerates the deposition of ECM in diverse cell types within in vitro culture systems [34], the most efficacious MMC agent for a particular cell type remains unclear. Accordingly, the present study examined the efficacy of two widely utilized molecules, carrageenan [108,157] and Ficoll™ [45,158], as MMC agents in the culture of T-LESCs. Our results indicated that both MMC agents increased ECM deposition, with the Ficoll™ cocktail (10 mg/mL Ficoll™ 70 kDa + 25 mg/mL Ficoll™ 400 kDa + 2.25 mg/mL Ficoll™ 1000 kDa) inducing significantly higher ECM deposition compared to carrageenan in T-LESC culture. Although carrageenan has been demonstrated to enhance ECM deposition more effectively than Ficoll™ cocktails [109,159], a recent study showed that a Ficoll™ cocktail (37.5 mg/mL Ficoll™ 70 kDa + 25 mg/mL Ficoll™ 400 kDa) induced higher collagen IV deposition than carrageenan in equine adipose-derived stem cell cultures [158]. Moreover, another recent study demonstrated that a Ficoll™ cocktail (10 mg/mL Ficoll™ 70 kDa + 25 mg/mL Ficoll™ 400 kDa + 1 mg/mL Ficoll™ 1000 kDa) induced higher collagen IV deposition than the −MMC group in human tenocyte cultures in the presence of fetal bovine serum (FBS) [160]. Additionally, in the same report, the Ficoll™ cocktail induced higher collagen IV deposition than carrageenan at day 5 in the presence of human serum. In human corneal fibroblast cultures, a Ficoll™ cocktail (37.5 mg/mL Ficoll™ 70 kDa + 25 mg/mL Ficoll™ 400 kDa) did not induce fibronectin deposition in the presence of newborn calf serum [161]. Conversely, in the same report, the use of the Ficoll™ cocktail resulted in enhanced fibronectin and collagen IV deposition when the human serum was used as a culture additive in the corneal fibroblasts. With regard to laminin, a Ficoll™ cocktail (37.5 mg/mL Ficoll™ 70 kDa + 25 mg/mL Ficoll™ 400 kDa) induced higher laminin deposition than the −MMC group in human bone marrow mesenchymal stromal cell cultures in the presence of FBS [162]. However, in the same report, no differences in laminin deposition were observed between the carrageenan and the Ficoll™ cocktail groups. A consideration of the existing literature suggests that the deposition of a specific ECM molecule is dependent on the MMC agent used, with the effect varying depending on the cell type in question. Furthermore, the potential influence of cell culture additives, such as FBS and human serum, on ECM deposition cannot be discounted. It should be noted that a serum-free culture medium was employed in the course of our study. An additional important perspective is that of the orientation of the ECM in the context of corneal bioengineering, given that the native corneal stroma displays aligned fibril formation [163]. Previous studies have reported that Ficoll™ induced aligned ECM deposition [164], whereas carrageenan induced globular ECM deposition [46]. Although our study did not provide evidence regarding the orientation of deposited ECM, it is imperative to develop cell culture substrates with an optimal structure and integrity for the target tissue. It is also noteworthy that, in addition to its use as a MMC agent, Ficoll™ is employed widely in the purification of stem cells, which have been utilized in clinical settings [165,166].

Consequently, as FC was observed to induce more effective ECM deposition in T-LESCs culture on TCP in the current study, only FC was employed as the MMC agent in the subsequent phase of the study.

### 4.3. Efficacy of Collagen Films in T-LESC Cultures in the Presence of MMC

The biological response of T-LESCs to collagen films fabricated in the current study was analyzed, and the data demonstrated that no notable differences were detected between the groups with regard to cellular viability and metabolic activity. Cell growth was shown to be supported by each collagen film fabricated. These observations indicate that the 4SP, the cross-linking agent used in the current study, is not cytotoxic to the cells, in accordance with the outcomes of previous studies in this field [132,133,134,135]. As expected, the collagen films yielded a higher degree of cell proliferation in comparison to TCP. This is an unsurprising observation since collagen is an essential constituent of the ECM and performs a crucial role in supporting critical cell functions such as adhesion, proliferation, and migration. In addition, the highest cell proliferation was observed in HA-enriched collagen films at each time point. This observation is particularly noteworthy when one considers that HA is one of the major constituents of the LESC niche, which plays an essential role in maintaining LESCs in their stem cell state [79,82]. It has been consistently demonstrated in previous studies that scaffolds incorporating HA [167] or culture substrates enriched with HA [168] facilitate the proliferation of LESCs in vitro. HA has been employed in varying proportions (e.g., 0.1%, 0.5%, and 3%) as a component in culture substrates designed for in vitro LESC culture [168,169], including our study. Although this appears to be a limitation of our study during the collagen-based film fabrication process, there is currently no consensus on the optimal HA concentration for in vitro LESC cultures due to the fact that the exact composition of the limbal niche has not yet been fully identified [82]. It is also crucial to underscore that high molecular weight (>500 kDa) HA exerts anti-inflammatory actions and is predominantly associated with tissue integrity. Conversely, low molecular weight (<200 kDa) HA has been linked to pro-inflammatory effects and is more closely related to pathogenesis [170,171]. Accordingly, a 1000 kDa molecular weight form of HA was employed in our study.

Continuing with the effect of MMC on T-LESCs cultured on collagen films, immunofluorescence results showed that MMC (Ficoll™ cocktail; FC) induced the deposition of ECM components (collagen type IV, fibronectin laminin), independent of the culture substrates (TCP, CF, and CF-HA) tested. Thus, the integration of collagen IV, fibronectin, and laminin into the HA-enriched collagen films through the self-assembly tissue engineering approach, enabled by the utilization of MMC, resulted in the generation of biomimetic substrates with augmented characteristics. Further immunofluorescence analysis for PAX6, vimentin, and CK12 (i.e., corneal cell markers [172,173,174]) showed no differences between the groups at any time point. Even though the cells used in the current study were immortalized, the lack of phenotypic changes throughout the cell culture process indicates that the methodology employed in the study did not induce phenotypic drift of the cells, as demonstrated by the preliminary analyses.

Indeed, donor tissue is currently the primary source of cells that meet the needs of regenerative medicine. A variety of cell types are extracted from donor material and expanded directly using primary culture techniques used in regenerative medicine [175,176,177,178,179]. However, this approach presents a number of significant challenges. These include the limited availability of cells due to the process of cellular senescence, in which the properties of the cells change after a certain number of divisions, and their heterogeneity due to different donors, different tissues, and different cell isolation and culture techniques [48]. The heterogeneity of cells severely limits the comparability of experimental and clinical results, both between different institutions and within the same laboratory setting. By contrast, immortalized cells, due to their increased expansion capacity and homogeneity [55,56,57,58], present a potential solution to these issues and offer a standardizable, scalable, sustainable, and consistent cell source for the manufacture of validated products [48,180]. Although immortalization has serious drawbacks, including the potential for malignant transformation or significant phenotypic alteration through prolonged culture or genetic modification [181,182], recent studies have proposed various strategies to enhance the biosafety of immortalized cells. These include CRISPR/Cas9-mediated immortal gene insertion, the utilization of decellularized ECM, and the incorporation of suicide genes to ensure post-transplant safety [183]. Moreover, clinical trials with immortalized cells are currently underway [184,185,186,187]. It is crucial to highlight that the concept presented in the current study does not involve the direct transplantation of immortalized cells into the human body. Rather, the objective is to enhance the deposition of ECM components produced and secreted by immortalized cells using MMC; however, safety validation of the concept is required through preclinical and clinical studies.

## 5. Conclusions

In recent years, cell-based tissue engineering approaches, including corneal bioengineering, have concentrated on increasing the biomimicry of the in vitro cell culture milieu, as the environment in which the cells are cultivated plays a key role in the determination of cell fate. This preliminary study demonstrates, for the first time, the potential of immortalized cells (T-LESCs), macromolecular crowding, and HA-enriched collagen-based films for the development of biomimetic substrates for limbal epithelial stem cell cultures. In particular, the use of macromolecular crowding with immortalized cells in place of primary cells allows for the deposition of tissue-specific ECM, which has the potential to facilitate the development of biomimetic supramolecular assemblies. In the future, following the successful decellularization of immortalized cells, the developed ECM-rich substrates in this study could serve as a potential alternative carrier for the therapeutic limbal cells used in corneal reconstruction in ophthalmology clinics. It is important to note that further assessments are necessary to substantiate the efficacy of the developed substrates, including mechanical and morphological evaluations of the substrates and characterization of therapeutic limbal cells expanded on the substrates. Overall, our approach to developing tissue-specific biomimetic substrates has the potential to contribute to the development of cell-based tissue engineering products and provide a novel perspective for researchers in the field of corneal bioengineering.

## Data Availability

Raw and processed data are available on reasonable request from Mehmet Gurdal.

## Supplementary Materials

The following supporting information can be downloaded at: https://www.mdpi.com/article/10.3390/life14121552/s1, Figure S1: Purity and quality assessment of extracted collagen type I. (A) SDS-PAGE of commercially available (standard) vs. in-house-extracted collagen type I; the distinctive electrophoretic mobility profile of collagen type I, indicated by the α1(I), α2(I), β and γ bands, was observed to be similar in both samples. (B) Collagen solubility assessment of commercially available (standard) vs. in-house-extracted collagen type I; no pellet was observed in either standard or in-house-extracted collagen samples following centrifugation. As collagen solubility assessment is performed by visual examination of the solutions (prior to and following centrifugation), it is regarded as a qualitative technique; Figure S2: Morphology (A), viability (B), metabolic activity (C), and DNA concentration (D) at days 4, 7, and 10 in T-LESC cultures on TCP without MMC (−MMC) and with MMC (FC and λ CR). The cellular metabolic activity was expressed as a % reduction in the alamarBlue^®^ and was normalized to the −MMC control group. T-LESCs: human telomerase-immortalized limbal epithelial stem cells; TCP: tissue culture plastic; MMC: macromolecular crowding; FC: Ficoll™ cocktail; CR: carrageenan. N = 3. Live cells: Green. Dead cells: Red. Scale bar: 100 µm; Figure S3: Indicative electrophoresis gels (A) and immunofluorescence images (B) of collagen type I at days 4, 7, and 10 in T-LESC cultures on TCP without MMC (−MMC) and with MMC (FC and λ CR). T-LESCs: human telomerase-immortalized limbal epithelial stem cells; TCP: tissue culture plastic; MMC: macromolecular crowding; FC: Ficoll™ cocktail; CR: carrageenan. STD: collagen type I standard. Collagen type I: Green. Nuclei: Blue. Scale bar: 100 µm; Figure S4: Immunofluorescence analysis of PAX6, vimentin, and CK12 at days 4, 7, and 10 in T-LESC cultures on TCP, CF, and CF-HA without MMC (−MMC) and in the presence of the FC (+MMC). T-LESCs: human telomerase-immortalized limbal epithelial stem cells; TCP: tissue culture plastic; CF: collagen films; CF-HA: collagen films enriched with HA; MMC: macromolecular crowding. PAX6, vimentin and CK12: Green. Nuclei: Blue. Scale bar: 100 µm; Figure S5: Complementary relative fluorescence intensity analysis of PAX6, vimentin, and CK12 normalized to cell number (%) at days 4, 7, and 10 in T-LESC cultures on TCP, CF, and CF-HA without MMC (−MMC) and in the presence of the FC (+MMC). T-LESCs: human telomerase-immortalized limbal epithelial stem cells; TCP: tissue culture plastic; CF: collagen films; CF-HA: collagen films enriched with HA; MMC: macromolecular crowding. N = 9.
