# Supplementary material for: Development of Biomimetic Substrates for Limbal Epithelial Stem Cells Using Collagen-Based Films, Hyaluronic Acid, Immortalized Cells, and Macromolecular Crowding
Source: Life (Basel). 2024 Nov 26;14(12):1552. doi: 10.3390/life14121552 (PMC11678493; doi:10.3390/life14121552)

Supplementary Information

Figure S1: Purity and quality assessment of extracted collagen type I. (A) SDS-PAGE of commercially available (standard) vs. in-house-extracted collagen type I; the distinctive electrophoretic mobility profile of collagen type I, indicated by the α1(I), α2(I), β and γ bands, was observed to be similar in both samples. (B) Collagen solubility assessment of commercially available (standard) vs. in-house-extracted collagen type I; no pellet was observed in either standard or in-house-extracted collagen samples following centrifugation. As collagen solubility assessment is performed by visual examination of the solutions (prior to and following centrifugation), it is regarded as a qualitative technique.

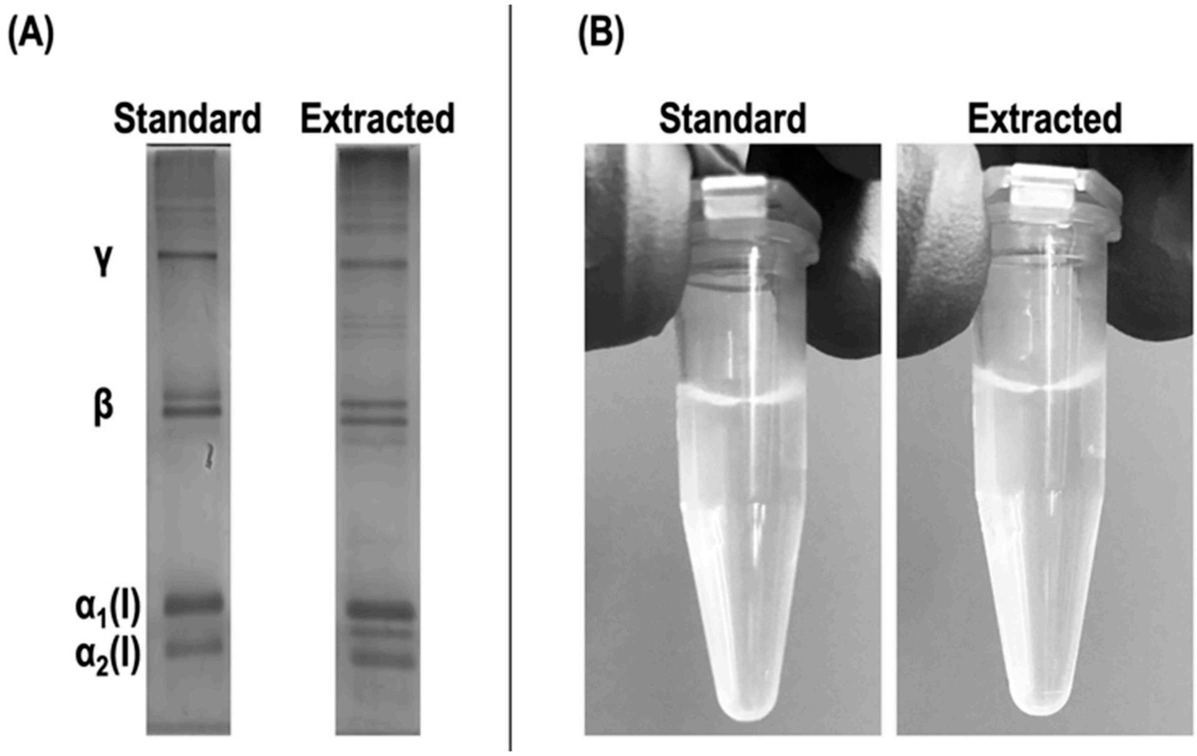

Figure S2: Morphology (A), viability (B), metabolic activity (C), and DNA concentration (D) at days 4, 7, and 10 in T-LESC cultures on TCP without MMC (−MMC) and with MMC (FC and λ CR). The cellular metabolic activity was expressed as a % reduction in the alamarBlue® and was normalized to the −MMC control group. T-LESCs: human telomerase-immortalized limbal epithelial stem cells; TCP: tissue culture plastic; MMC: macromolecular crowding; FC: Ficoll™ cocktail; CR: carrageenan. N = 3. Live cells: Green. Dead cells: Red. Scale bar: 100 μm.

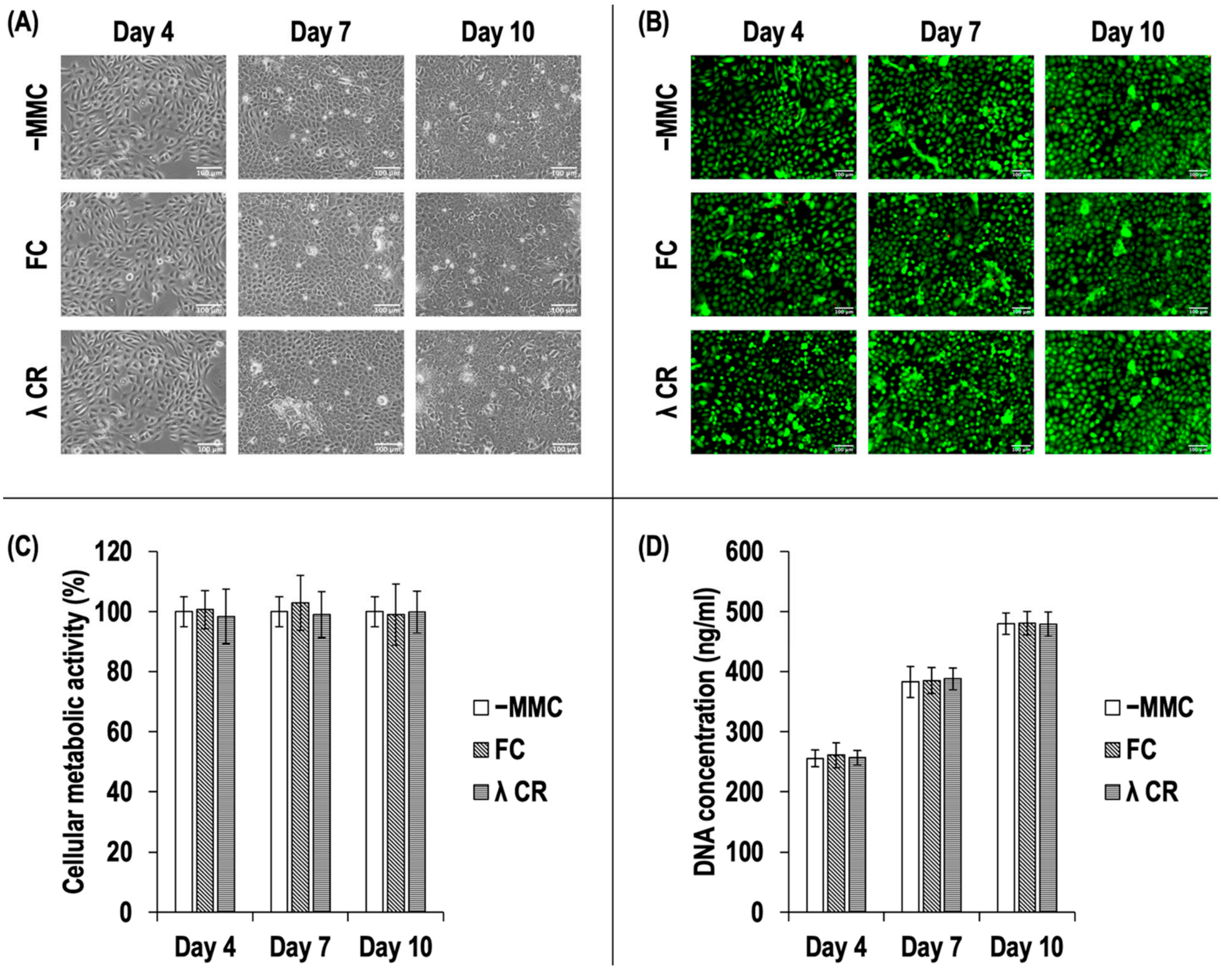

Figure S3: Indicative electrophoresis gels (A) and immunofluorescence images (B) of collagen type I at days 4, 7, and 10 in T-LESC cultures on TCP without MMC (−MMC) and with MMC

(FC and λ CR). T-LESCs: human telomerase-immortalized limbal epithelial stem cells; TCP: tissue culture plastic; MMC: macromolecular crowding; FC: Ficoll™ cocktail; CR: carrageenan. STD: collagen type I standard. Collagen type I: Green. Nuclei: Blue. Scale bar: 100 µm.

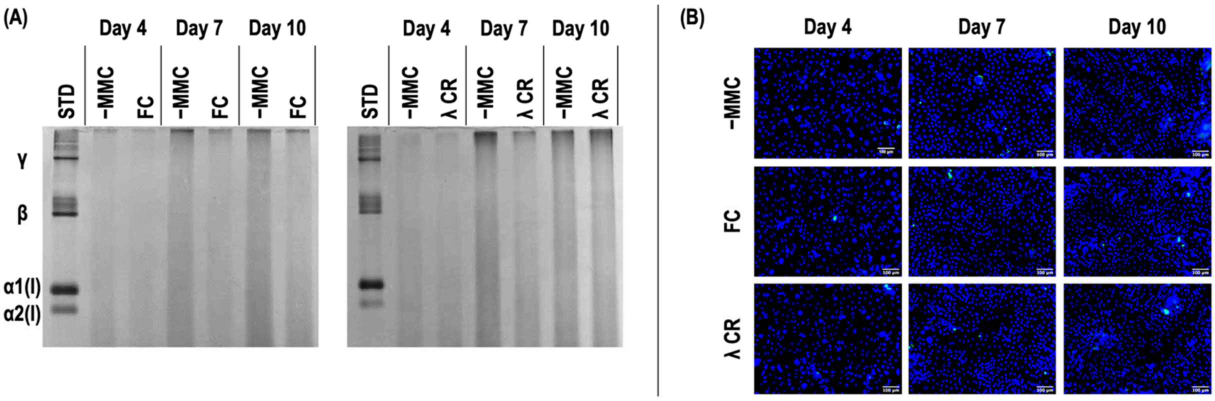

Figure S4: Immunofluorescence analysis of PAX6, vimentin, and CK12 at days 4, 7, and 10 in T-LESC cultures on TCP, CF, and CF-HA without MMC (−MMC) and in the presence of the FC (+MMC). T-LESCs: human telomerase-immortalized limbal epithelial stem cells; TCP: tissue

culture plastic; CF: collagen films; CF-HA: collagen films enriched with HA; MMC: macromolecular crowding. PAX6, vimentin and CK12: Green. Nuclei: Blue. Scale bar: 100 µm.

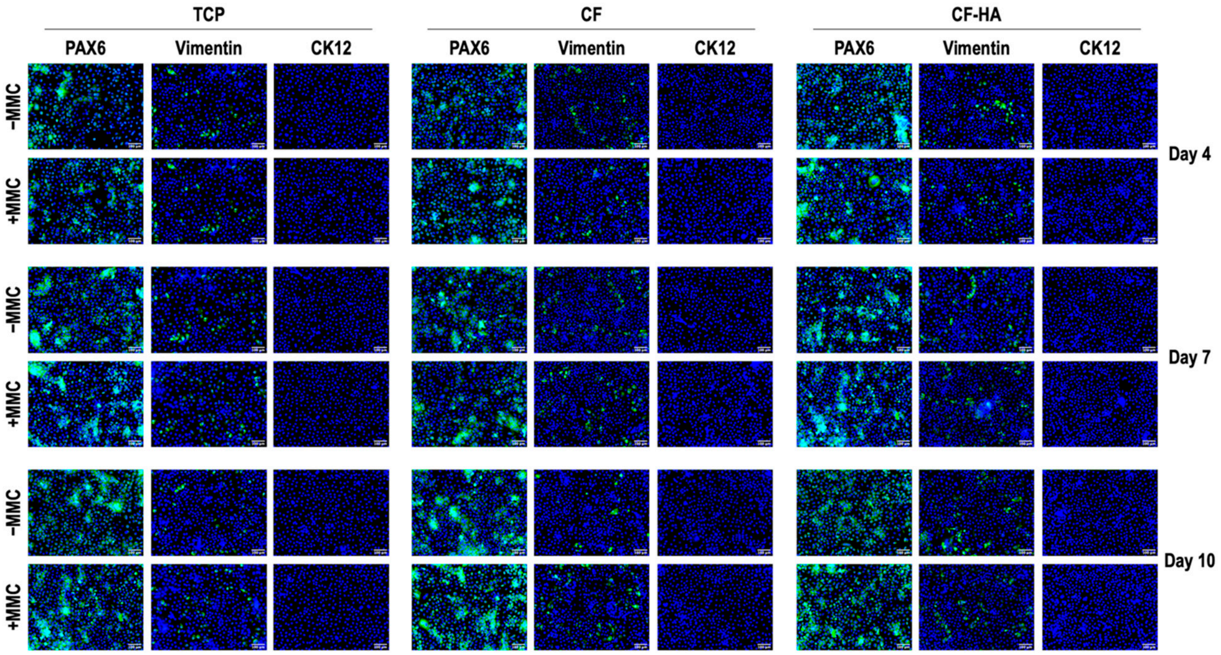

Figure S5: Complementary relative fluorescence intensity analysis of PAX6, vimentin, and CK12 normalized to cell number (%) at days 4, 7, and 10 in T-LESC cultures on TCP, CF, and CF-HA without MMC (−MMC) and in the presence of the FC (+MMC). T-LESCs: human telomerase-

immortalized limbal epithelial stem cells; TCP: tissue culture plastic; CF: collagen films; CF-HA: collagen films enriched with HA; MMC: macromolecular crowding. N = 9.

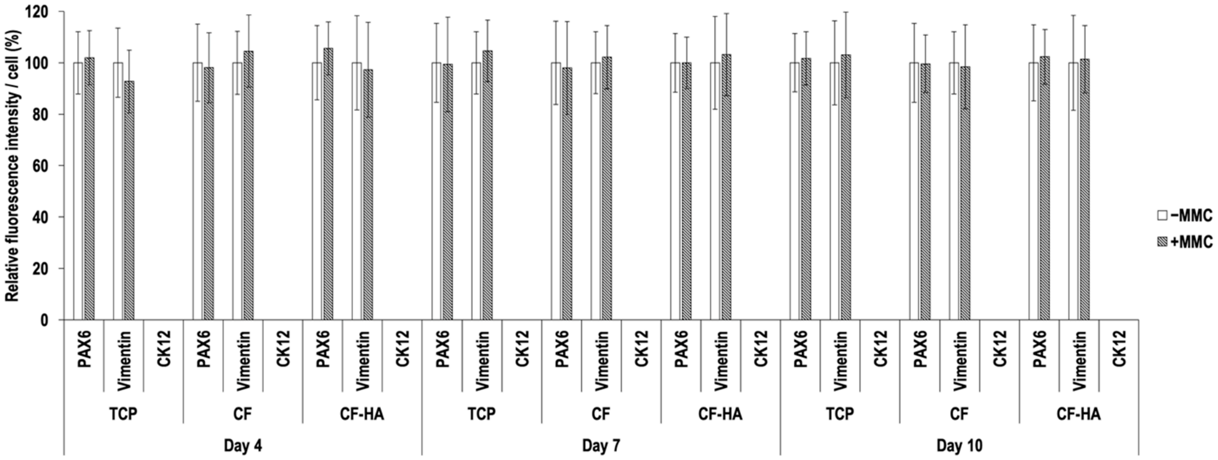

Supplement: Supplementary file 1 [file life-14-01552-s001.zip › life-3309279-supplementary.pdf]
